# The Impact of Comorbidities on Patient Outcomes in the Upper Body Lift: A Retrospective Review

**DOI:** 10.1093/asjof/ojac063

**Published:** 2022-07-28

**Authors:** Richard Cinclair, Zhiguo Shang, Al Aly, Jeffrey Kenkel

**Affiliations:** Department of Plastic Surgery, University of Texas Southwestern Medical Center, Dallas, TX, USA; Department of Bioinformatics, University of Texas Southwestern Medical Center, Dallas, TX, USA; Department of Plastic Surgery, University of Texas Southwestern Medical Center, Dallas, TX, USA; and a body contouring section editor for Aesthetic Surgery Journal; Department of Plastic Surgery, University of Texas Southwestern Medical Center, Dallas, TX, USA; editor-in-chief of ASJ Open Forum; and associate editor of Aesthetic Surgery Journal

## Abstract

**Background:**

Nonsurgical and surgical weight loss options have improved over the past several decades resulting in an increased number of patients who present with body contour deformities. This review focuses on the upper truncal deformity. This deformity is discernable by its residual tissue laxity in the upper arm, back, lateral chest, and breast.

**Objective:**

The purpose of this study is to evaluate the morbidity of this procedure when these regions are treated in one operative setting.

**Methods:**

A retrospective chart review of patients who underwent an upper body lift for truncal deformities after massive weight loss by the senior author between August 2006 and December 2019 was performed. Patient comorbidities and demographics, preoperative parameters, operative factors, and minor and major complications were assessed.

**Results:**

No intraoperative or major complications occurred. The overall complication rate was 71% (20/28), which were all minor and most related to wound breakdown. Using logistical regression analysis, we found that neither BMI nor amount of weight resected contributed to a higher complication rate in this cohort. Simple matching coefficients analysis identified anemia, hypertension, lifetime smoking history, celecoxib use, and multiple concurrent procedures as comorbidities and intraoperative factors with an increased risk for adverse outcomes.

**Conclusions:**

This review helps define the role of upper body lift in the care of patients with massive weight loss and addresses the morbidity of a comprehensive approach to upper body deformity. Appropriate patient selection, preoperative patient counseling, sound operative technique, and supportive postoperative care can help to avoid adverse outcomes.

**Level of Evidence: 4:**

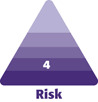

The age-adjusted prevalence of obesity (BMI > 30) and severe obesity (BMI > 40) were, respectively, 42.4% and 9.2% among adults in the United States in 2018.^1^ To address this epidemic, bariatric surgery has become a main stay of obesity treatment.^2^ The result is an increased number of patients who present with a variety of body contour deformities after massive weight loss.^3^

The lower trunk, which circumferentially spans from the inframammary (IMF) crease to the pelvis, is usually treated first after massive weight loss because this is the area of greatest concern for most patients. However, most massive weight loss patients also have complaints about their upper trunk and thighs.^4,5^ The upper truncal deformities are caused by overinflation of the skin/fat envelope from the inferior border of the neck to the IMF circumferentially, with subsequent deflation. The pattern and extent of deformity are based on the fat deposition pattern of any individual and the effects of the zones of adherence on the descent of the skin/fat envelope. The zones of adherence in the upper trunk are located over the sternum anteriorly and the spine posteriorly.^6^ These deformities to their full extent involve:

- Upper arm excess, with forearm involvement in some cases;
- Descent of the IMF, especially laterally;
- Upper back/lateral breast rolls/excess;
- Differing deformities of the breast or chest depending on the gender of the patient.

These deformities vary from patient to patient, but the key indications for an upper body lift procedure are upper back/lateral breast rolls/excess.^6^ These deformities almost universally occur together. A few patients may present with 1 or 2 of the components and, if so, can be treated in isolation.

It is the intent of this paper to focus on the overall complication rate associated with upper body lifts, whether patient characteristics or perioperative factors had an impact on complication rates and how this patient population’s risk factors affect the success of the operation, in a group of patients that one of the senior authors (J.K.) has operated on, over a 14-year period.

Upper body lift procedures are not all identical and will vary based on the gender and variable nature of the deformities as well as the philosophy of the surgeon. The goal of this study is not to describe the surgical technique/s utilized in upper body lifts. This will be covered in a separate article encompassing the variety between patients and the differences between the techniques utilized by the 2 senior authors.

## METHODS

We carried out a retrospective chart review of all upper body lift procedures performed consecutively between August 2006 and December 2019 by one of the senior authors, J.K., at a single institution after massive weight loss. Inclusion criteria were as follows: Weight stable for ≥6 months following weight loss mechanism; upper body lift as the primary procedure; upper truncal excess with inverted “V” deformities/upper back excess; nicotine free at the time of surgery; American Society of Anesthesiologists (ASA) I, II, or III; well-controlled medical comorbidities; a preoperative medical clearance evaluation; and an understanding of postoperative complications and scarring. All patients who underwent upper body lift procedures in a single operation were included in the chart review. One patient was excluded from the study for having the upper body lift performed in a staged manner. Waiver for informed consent was obtained, as per IRB protocol, as all information collected was de-identified. All charts were reviewed in depth by a single investigator for reliability. This study was performed in accordance with the IRB.

Data on patient characteristics, comorbidities, medications, intraoperative parameters, and complications were collected. Patient characteristics included gender, age, BMI, and ASA status. Patient comorbidities included diabetes, hypertension, anemia, coronary artery disease, cancer, renal disease, lifetime history of smoking, previous 30-day history of smoking, history of deep vein thrombosis, thyroid status, and asthma. Intraoperative parameters included liposuction performed, concomitant procedure, total mass resected per operation, and pain control technique. Complications evaluated were infection, superficial wound, deep wound, erythema, fat and skin necrosis, seroma, hematoma, further necessary procedures, and time after surgery of presentation of adverse events.

Three statistical analyses were conducted to identify items of significance as well as correlations. A Fisher’s exact *t*-test was applied to calculate the significance between patient characteristics and outcomes. A logistical regression was used to test correlation between BMI and weight resected with outcomes. Finally, simple matching coefficients (SMCs) were used to calculate the correlation between patient characteristics and outcomes.

### Operative Technique and Postoperative Protocol

Briefly, the female breast requires a thorough clinical analysis focusing specifically on the degree of deflation with associated skin laxity, how much breast tissue remains, and nipple-areola position. These factors contribute to suggested surgical options that may include: breast augmentation, mastopexy with or without an implant, and breast reduction. For simplicity, this technique will focus on the male upper body lift as the female breast presents with a wider variety of deformities in this population.

**Figure 1.**
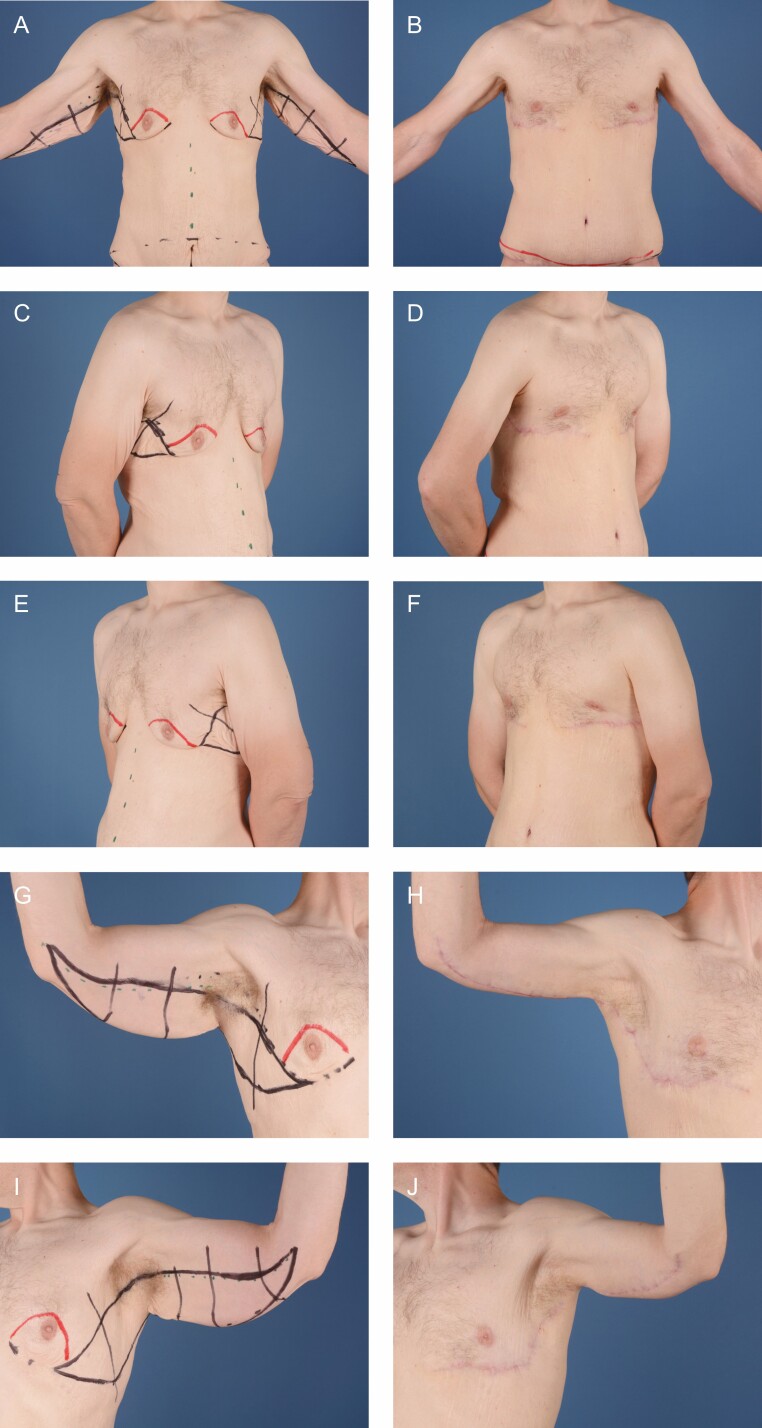
A 39-year-old male patient (A, C, E, G, I) before and (B, D, F, H, J) 7 months photographs after an upper body lift, shown in portrait view, right oblique view, left oblique view, view of right axilla, and view of left axilla, respectively.

**Figure 2.**
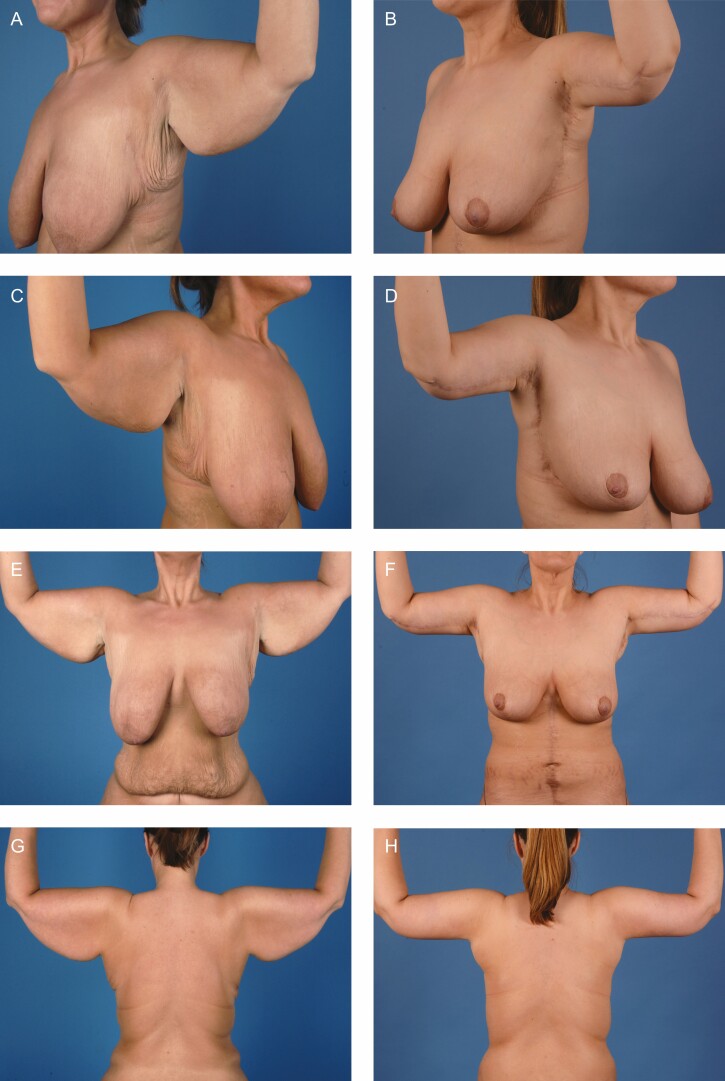

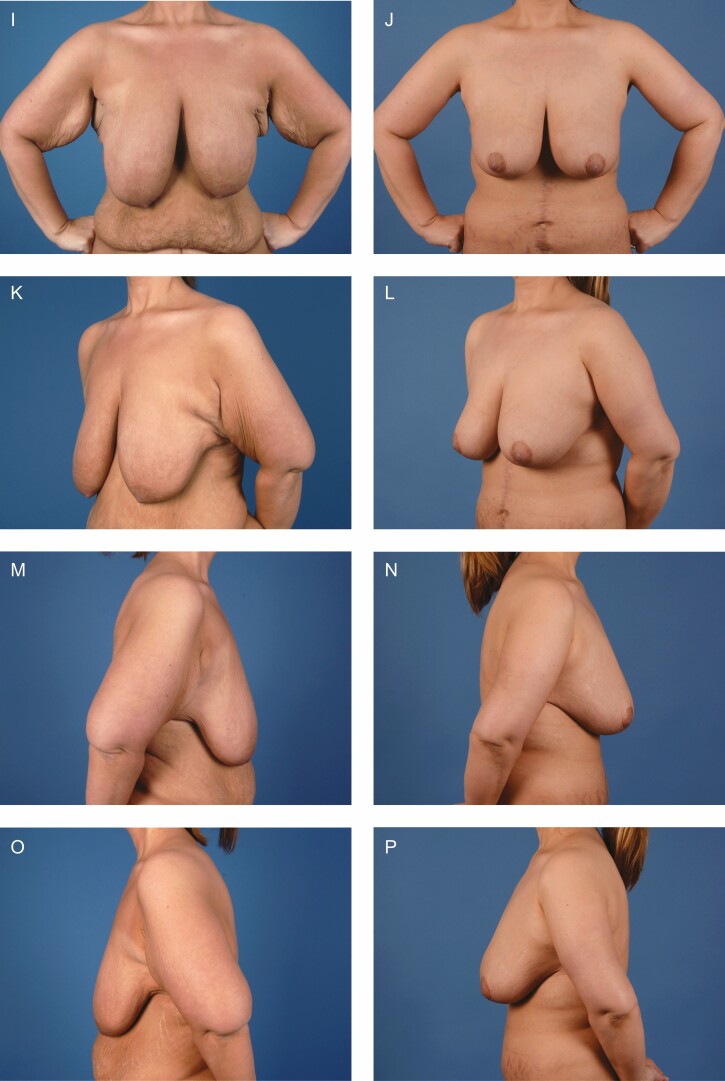
A 34-year-old female patient shown (A, C, E, G, I, K, M, O) before and (B, D, F, H, J, L, N, P) 2 years after an upper body lift: view of left axilla, view of right axilla, portrait view arms raised, back view arms raised, portrait view, oblique view, right side profile view, and left side profile view, respectively.

The patient is positioned supine with the arms suspended and secured overhead. A standard wetting solution of lactated ringers and Epinephrine is then infiltrated into the arms, lateral chest, and upper chest. The operation begins with a focus on the arms. An upper incision is made retrograde from the elbow to the axilla and carried down through Scarpa’s fascia. Then, undermining is performed posteriorly in this plane to the predetermined posterior marking. Care is taken in the distal third of the upper arm to identify and preserve the Median Antebrachiocutaneous nerve. Once hemostasis is achieved, the skin is tailor tacked and marked to be resected. It is resected distally to proximally and approximated using staples. Then, the superficial fascia is approximated using 2-0 and 3-0 absorbable barbed sutures. Finally, the arms are returned to 90 degrees and secured. In males, SAFE liposuction is performed, and the anterior chest is debulked.^7^ Next, the anterior chest flap is deepithelialized and elevated with care taken to ensure the preservation of the subdermal plexus. Once this is completed, excess breast tissue is resected, and the upper flap is undermined. Continuity is then gained from the axilla down through the lateral chest and the lateral flap is elevated and released anterior and lateral to the *latissimus dorsi* muscle. Once hemostasis is achieved, the IMF crease is approximated with 0 braided absorbable suture, 2-0 absorbable monofilament, 3-0 absorbable monofilament, and 4-0 absorbable barbed suture. The anterior border of the chest is secured using 0 braided absorbable suture. Next, the lateral skin is tailor tacked, placing the inferior flap under tension, and secured to the chest wall with 0 PDS. The remainder of the incision is closed in a similar manner. Finally, the patient is sat up and the nipple position is marked at the 4^th^ intercostal space along the lateral border of the *pectoralis* muscle. It is deepithelialized and a cruciate incision is made to facilitate delivery of the nipple. The nipple is inset using 4-0 absorbable monofilament and 4-0 absorbed barbed suture. The patient is then placed in a supportive garment and awoken. Briefly, in females, it is imperative to shape the breast and establish the breast mound before any excision laterally as this will occupy some of the lateral skin. If resection is done before breast shaping, the breast mound will be displaced laterally. Preoperative, marking, and postoperative photos are shown in Figures 1A-J and 2A-P.

## RESULTS

The study included a total of 28 patients. Patient characteristics are displayed in Table 1. There were 22 females and 6 males. The average age was 43.5 years (range, 22-64 years) with an average weight loss of 165 lbs (range, 60-410 lbs.). The average BMI (kg/m^2^) at the time of plastic surgery was 30.6 (range, 21.2-39), with most patients categorized as either overweight or obese. The most common method of weight loss was Roux-en-Y gastric bypass, followed by gastric sleeve and diet and exercise. Twenty-three patients (82%) had a concomitant body contouring procedure with abdominoplasty being the most common followed by thigh lift as displayed in Table 2. The average operating time was 351 minutes (range, 257-477 min). Patient comorbidities are displayed in Table 3, with the most common being hypertension (25%). The number of patient comorbidities was tallied, and most patients presented with no comorbidities (50%) followed by 2 comorbidities (21%). Patient follow-up was 25 months on average (range, 1-120 months).

**Table 1.** Patient Characteristics

Variable	N	%
Gender		
Female	22	78.6
Male	6	21.4
Age (years)		
<30	5	17.9
30-39	5	17.9
40-49	9	32.1
≥50	9	32.1
BMI (kg/m^2^)		
Normal (<25)	2	7.1
Overweight (≥25)	12	42.9
Obese (≥30)	9	32.1
Severely obese (≥35)	5	17.9
Morbidly obese (≥40)	0	0.0
Weight loss method		
Roux-en-Y bypass	11	39.3
Gastric sleeve	6	21.4
Lap band	4	14.3
Diet & exercise	6	21.4
Duodenal switch	1	3.6
Amount of weight lost (lbs)		
50-99	3	10.7
100-149	10	35.7
150-199	8	28.6
200-249	3	10.7
>250	4	14.3
ASA status		
ASA I	5	17.9
ASA II	22	78.6
ASA III	1	3.6
Pain control		
Pain pump	13	46.4
Exparel infiltration	13	46.4
TAP block with exparel	2	7.1
Total operative time (min)		
200-299	6	21.4
300-399	17	60.7
400-499	5	17.9

ASA, American Society of Anesthesiologists; TAP, transversus abdominis plane.

**Table 2.** Concomitant Procedures

Procedure	N (%)
Patients with a concomitant procedure	23 (82.1)
Abdominoplasty	18 (78.3)
Thigh lift	4 (17.4)
Neck lift	2 (8.7)
Explant	1 (4.3)
Panniculectomy	3 (13.0)

**Table 3.** Patient Comorbidities

Variable	N (%)
Hypertension	7 (25.0)
Diabetes	1 (3.6)
Coronary artery disease	0
Anemia	2 (7.1)
Renal disease	0
Cancer	0
History of DVT/PE	1 (3.6)
Ever Smoked	3 (10.7)
Hypothyroidism	6 (21.4)
Other PMHx	16 (57.1)
Number of comorbidities	
0 1 2 3 4 5+	14 (50.0) 5 (17.9) 6 (21.4) 1 (3.6) 0 2 (7.1)

DVT, deep vein thrombosis; PE, pulmonary embolism; PMHx; past medical history.

No intraoperative or major complications occurred. The overall complication rate was 71% (20/28), which were all minor and most related to wound breakdown. As presented in Table 4, 16/28 (57%) patients experienced any kind of wound breakdown. Seven patients required revisionary surgery, most commonly scar revision secondary to difficulties with wound healing.

**Table 4.** Patient Outcomes

Complication	N (%)
Infection	5 (17.9)
Wound breakdown	16 (57.1)
Revisions	7 (25.0)
Erythema	1 (3.6)
Seroma	2 (7.1)
Hematoma	0
Necrosis	2 (7.1)

**Figure 3.**
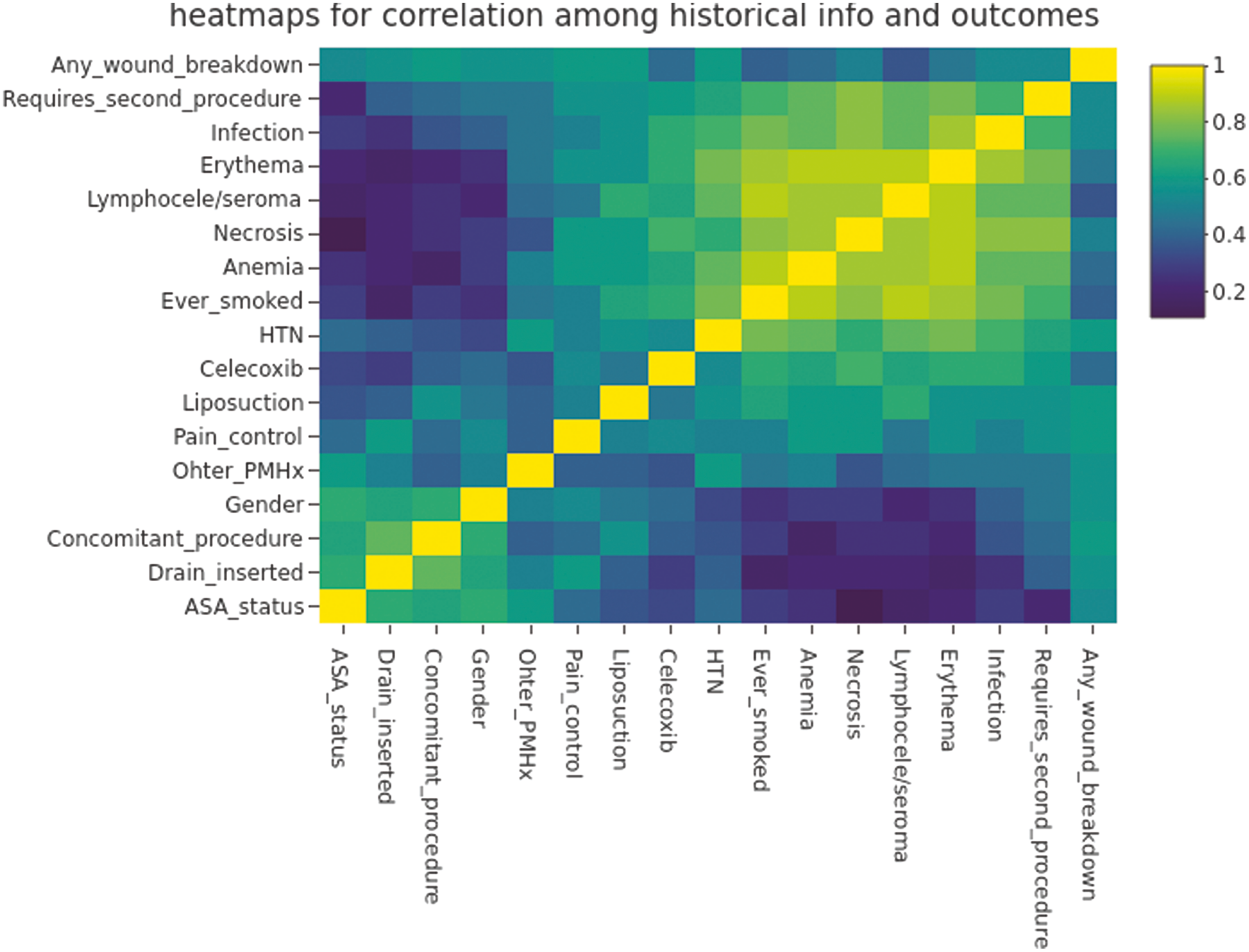
Heat map of simple matching coefficient shows correlation between historical characteristics and outcomes.

Using logistical regression analysis, we found that neither BMI nor amount of weight resected contributed to a higher complication rate in this cohort (Table 5). Our Fisher’s exact *t*-test analysis found a significance between the ASA classification and the complication of postoperative necrosis (Table 6). Finally, the SMC analysis showed that anemia was highly correlated with risk for the complications of necrosis, erythema, or infection in this cohort (Figure 3). In addition, it also showed hypertension as a risk factor highly correlated with the incidence of erythema and infection. We found that ever having smoked correlated with a higher risk for seroma formation. Finally, we found that ever having smoked, undergoing a concomitant procedure, and celecoxib use intraoperatively were all associated with an increased risk of any wound breakdown.

**Table 5.** *P*-values of Logistic Regression Test of BMI and Weight Resected With Outcomes

Variable	Any wound breakdown	Erythema	Necrosis	Lymphocele/seroma	Requires second procedure	Infection
BMI (kg/m^2^)	0.671	0.262	0.848	0.371	0.39	0.762
Weight resected	0.764	1	0.835	0.173	0.174	0.193

**Table 6.** *P*-value of Fisher’s Exact Test of Historical Characteristics With Outcomes

Variable	ASA status	Drain inserted	Concomitant procedure	Gender	Other_PMHx	Pain control	Liposuction	Celecoxib	Hypertension	Ever smoked
Necrosis	0.026	N/A	N/A	N/A	0.175	0.206	N/A	0.497	N/A	N/A
Lymphocele/seroma	0.331	N/A	N/A	0.389	N/A	0.484	0.146	N/A	0.444	0.206
Erythema	N/A	N/A	N/A	N/A	N/A	0.464	N/A	N/A	0.25	N/A
Infection	N/A	N/A	0.55	0.553	N/A	N/A	N/A	0.606	0.574	0.459
Requires second procedure	0.082	0.545	0.29	0.288	N/A	0.67	N/A	N/A	N/A	N/A
Any wound breakdown	N/A	N/A	0.624	N/A	0.702	0.276	0.253	0.691	0.184	0.56

ASA, American Society of Anesthesiologists; N/A, not applicable; PMHx, past medical history.

## Discussion

The main purpose of this study was to better understand how patient characteristics and perioperative factors have an impact on patient outcomes. Although our study is limited by sample size, several factors were found to have significant effects on outcomes. First, we did not observe BMI, or the amount of tissue resected, contributing to higher complications in this cohort. This finding is consistent with that of a previous study by Bunting et al where no significance was found between BMI and complication rates in post massive weight loss patients who underwent vertical abdominoplasty.^3^ This study concurs with a previous study by Langer et al on the lack of risk associated with BMI in body contouring procedures.^3,8,9^ This suggests that this procedure is safe for patients with varying BMIs and necessary resections. We believe that BMI should not solely preclude a patient from an upper body lift. Nevertheless, it does differ significantly from the findings in other body contouring studies by Greco et al and Borud and Warren.^10,11^ Although, it is neither confirmed nor denied by this study, BMI does play an important role in that a patient should be at a stable BMI for at least 6 months before surgery for the most predictable outcomes.

The ASA grading system stratifies patients based on their operative risks.^12^ Miller et al showed that in plastic surgery operations, high-risk ASA greater than III significantly increased the odds of venous thromboembolism (odds ratio = 4.17). In addition, the higher-risk group had a higher incidence of infection and delayed wound healing.^13^ Importantly, all patients except one were less than or equal to ASA II. The Fisher’s exact *t*-test revealed statistical significance between ASA classification and the complication of postoperative necrosis. Unexpectedly, both patients who experienced necrosis in this cohort were both ASA I. One patient had an associated infection, while the second patient had no other associated complications. The scarcity of necrosis as an outcome limits the reliability of this result significantly. Other body contouring studies that have previously evaluated ASA status have not found any significance in complication rates.^3,14^

SMC analysis offered insight into some patient characteristics that are correlated with higher incidences of complications. Nutritional status is important for wound healing and postsurgical recovery. In the same manner, it is important that tissues receive adequate oxygenation to undergo the healing process. In our cohort, we saw anemia and the complications of necrosis and infection to be highly correlated. We had two cases of necrosis: one case had bilateral skin necrosis in the breasts associated with a nonhealing wound secondary to infection while the second case experienced fat necrosis worse in the left breast than the right. In general, post-bariatric surgery patients can struggle with anemia based on iron availability and other mechanisms.^15^ A meta-analysis by Weng et al. found the prevalence of anemia 24 months after bariatric surgery to be 25.9%.^16^ It is worth noting that while previous studies have acknowledged the role of screening for anemia in post-bariatric surgery patients, none have reported anemia as a risk factor for postoperative complications in body contouring. Approximately, 25% of the vertical abdominoplasty patients with a complication in the study by Bunting et al presented with anemia. With up to a quarter of post massive weight loss patients presenting with anemia, this is a risk factor that deserves proper attention particularly if multiple body contouring procedures are in one setting. Further studies should be undertaken to investigate to what effect the degree of anemia has on the risk of complications.

We also found that hypertension is a comorbidity to be cognizant of. Previously published body contouring data have mixed results on the effects of hypertension as a risk factor for complications.^17,18^ Importantly, in our cohort, hypertension was the most recorded comorbidity; 7 of the 7 patients who presented with hypertension also had other past medical history that was not directly evaluated in this study due to the limited sample size of these conditions. This past medical history included but was not limited to: medication allergies, gastroesophageal reflux, psychological diagnoses, obstructive sleep apnea, and product allergies. Given that in this study hypertension presents with comorbid conditions, it is difficult to conjecture that hypertension alone increases the risk for complications. Thus, a patient’s complete medical history should be considered and well-controlled before an upper body lift.

Although we did not operate on any active smokers, we did still find an increased risk of seroma formation among patients with a lifetime history of smoking. These findings are corroborated by a previous body contouring study by Bunting et al.^3^ This suggests that even patients who have quit smoking should be counseled on the potential risk of minor postoperative complications. Ideally, smokers should quit 4 to 8 weeks before surgery and not smoke for 4 to 6 weeks after surgery.^19-21^

Wound breakdown is a well-established complication in body contouring procedures and our cohort followed this trend.^3,8,17,18^ Any wound breakdown, regardless of size or severity, was considered a complication, which likely contributed to our reported higher complication rate. Complete wound dehiscence, down to the level of the underlying muscle fascia, was not observed. All the wound breakdown complications were superficial and minor in character. Given the post massive weight loss quality of the skin, wound breakdown is not a surprising complication and was by far our most common. Our study identified the risk factors of ever having smoked, undergoing a concomitant procedure, and intraoperative celecoxib use as factors of concern when considering possible wound breakdown. Currently, animal models report contradictory evidence on the effect of selective COX-2 inhibition on wound healing.^22-24^ No human studies have reported on this relationship previously. Winocour et al and Bunting et al have also shown an increased risk of wound breakdown in association with multi-body contouring procedure operations.^3,25^ We believe that this risk can be mitigated to an extent by achieving a tension-free closure, preoperative counseling, and adequate nutrition.

Counseling a patient appropriately is imperative to the surgeon-patient relationship as well as establishing appropriate patient expectations for potential complications and outcome. In the upper body lift, is it particularly important that the patient understands the postoperative scar burden. In addition, these findings emphasize the importance of a multidisciplinary approach to these patients in terms of appropriate medical therapies, BMI stabilization, and optimized nutrition. When these areas are neglected or our massive weight loss populations’ risk factors go unacknowledged, patients and surgeons can experience dissatisfaction, an increased risk of complications, and less than optimal aesthetic outcomes.

The small sample size in this study and its retrospective nature limit some of the reliability of the data. Additionally, while SMC analysis is able to show correlations between data, it is limited in its ability to describe causation. Further studies should aim to measure the significance of the severity of a risk factor to postoperative outcomes.

## Conclusions

The upper body lift is a combination of a brachioplasty, lateral chest excision, and breast shaping procedure. Our data show that this is a safe procedure with predictable outcomes. Although our complication rate was high, the overwhelming majority were minor in the form of wound healing problems and infection. To avoid potential complications, the specific risk factors to be aware of when assessing a patient for an upper body lift include anemia, arterial hypertension, and a positive lifetime history of smoking. The decision to proceed with multiple body contouring operations in the same procedure should be weighed against the increased risk of wound breakdown. Importantly, BMI and the amount of mass resected do not play a role in the risk of postoperative complications. This study affords us a better understanding of the risk profile of our massive weight loss patient population in the upper body lift. Nevertheless, this is a small case series, and more research on the topic should be conducted in the future. This series underlines the steps plastic surgeons can take to keep patients safe and minimize complications in the upper body lift.
